# Highly multiplexed quantifications of 299 somatic mutations in colorectal cancer patients by automated MALDI-TOF mass spectrometry

**DOI:** 10.1186/s12920-020-00804-y

**Published:** 2020-10-02

**Authors:** Chang Xu, Danli Peng, Jialu Li, Meihua Chen, Yujie Hu, Mingliang Hou, Qingjuan Shang, Qi Liang, Jie Li, Wenfeng Li, Xiaoli Wu, Changbao Liu, Wanle Hu, Mao Cai, Huxiang Zhang, Guorong Chen, Lingling Yu, Xiaoqun Zheng, Feizhao Jiang, Ju Luan, Shengnan Jin, Chunming Ding

**Affiliations:** 1 Department of Colorectal and Anal Surgery, The First Affiliated Hospital of Wenzhou Medical University, Wenzhou, Zhejiang Province China; 2 School of Laboratory Medicine and Life Sciences, Wenzhou Medical University, Wenzhou, Zhejiang Province China; 3 Key Laboratory of Laboratory Medicine, Ministry of Education, Wenzhou Medical University, Wenzhou, Zhejiang Province China; 4 Department of Radiotherapy and Chemotherapy, The First Affiliated Hospital of Wenzhou Medical University, Wenzhou, Zhejiang Province China; 5 Department of Gastroenterology, The First Affiliated Hospital of Wenzhou Medical University, Wenzhou, Zhejiang Province China; 6 Department of Coloproctology, The Second Affiliated Hospital and Yuying Children’s Hospital of Wenzhou Medical University, Wenzhou, Zhejiang Province China; 7 Department of Pathology, The First Affiliated Hospital of Wenzhou Medical University, Wenzhou, Zhejiang Province China; 8 Department of Laboratory Medicine, The Second Affiliated Hospital and Yuying Children’s Hospital of Wenzhou Medical University, Wenzhou, Zhejiang Province China

**Keywords:** Somatic mutation, Colorectal cancer, MALDI-TOF mass spectrometry, Multiplex detection

## Abstract

**Background:**

Detection of somatic mutations in tumor tissues helps to understand tumor biology and guide treatment selection. Methods such as quantitative PCR can analyze a few mutations with high efficiency, while next generation sequencing (NGS) based methods can analyze hundreds to thousands of mutations. However, there is a lack of cost-effective method for quantitatively analyzing tens to a few hundred mutations of potential biological and clinical significance.

**Methods:**

Through a comprehensive database and literature review we selected 299 mutations associated with colorectal cancer. We then designed a highly multiplexed assay panel (8-wells covering 299 mutations in 109 genes) based on an automated MADLI-TOF mass spectrometry (MS) platform. The multiplex panel was tested with a total of 319 freshly frozen tissues and 92 FFPE samples from 229 colorectal cancer patients, with 13 samples also analyzed by a targeted NGS method covering 532 genes.

**Results:**

Multiplex somatic mutation panel based on MALDI-TOF MS detected and quantified at least one somatic mutation in 142 patients, with *KRAS*, *TP53* and *APC* being the most frequently mutated genes. Extensive validation by both capillary sequencing and targeted NGS demonstrated high accuracy of the multiplex MS assay. Out of 35 mutations tested with plasmid constructs, sensitivities of 5 and 10% mutant allele frequency were achieved for 19 and 16 mutations, respectively.

**Conclusions:**

Automated MALDI-TOF MS offers an efficient and cost-effective platform for highly multiplexed quantitation of 299 somatic mutations, which may be useful in studying the biological and clinical significance of somatic mutations with large numbers of cancer tissues.

## Background

The cancer genomes carry many different genomic alterations including somatic mutations. Most notably, driver mutations are causally implicated in oncogenesis. They have conferred a growth advantage on the cancer cells [1]. Increasing number of mutations are being characterized for better treatment selection and prognosis [2]. In non-small cell lung cancer, for example, patients with epidermal growth factor receptor (*EGFR*) mutation are likely to benefit from tyrosine kinase inhibitors (TKIs) [3]. On the opposite, colorectal cancer patients with *KRAS* or *NRAS* mutations should not be treated with either *EGFR*-inhibitor Cetuximab [4] or Panitumumab [5].

Several methods are available for the detection of tumor somatic mutations. Next generation sequencing (NGS) is widely adopted as a discovery tool for somatic mutation analysis in cancer [6]. Using NGS, The Cancer Genome Atlas (TCGA) presents genomic alternations identified from over 11,000 tumor samples from 33 cancer types, providing valuable insights into new targets for drug development, treatment selection, or even combination therapies for personalized medicine [7]. While NGS methods can analyze hundreds to tens of thousands mutations [8], methods such as qPCR or digital PCR are ideal for analyzing a few clinically important mutations with high efficiency and cost-effectiveness [9, 10]. However, as biologically and clinically important mutations for many cancer types continue to be identified, there is a growing unmet need to cost-effectively and quantitatively analyze tens to a few hundred of mutations, in highly heterogenous cancer tissues where mutant allele frequency can be highly variable.

The MassARRAY platform, based on automated MALDI-TOF mass spectrometry, is suitable to meet this growing need due to its high multiplex capability, flexibility for both sample size and mutation number, quantification capability, and automation in sample processing and data analysis [11]. The MassARRAY can detect up to 40 mutations in a single well on a 96 or 384-well plate.

In this report, we developed and validated an 8-well panel covering 299 most common somatic mutations in colorectal cancer (CRC). Such multiplex level may be sufficient for detecting virtually all clinically relevant mutations in a cancer type, with cost-effectiveness and turnaround time desirable in real-life clinical settings.

## Method

### Patients recruitment

Patients diagnosed with colorectal cancer were recruited with informed consent between July 2015 and June 2019 from the First Affiliated Hospital and the Second Affiliated Hospital of Wenzhou Medical University, China. Exclusion criteria include more than two pathological types, metastasis, treated with neoadjuvant chemotherapy or immunotherapy before surgery, or any cancer within the past 5 years. Tumor stages were determined according to the 8th edition of the American Joint Committee on Cancer (AJCC). The study was approved by the ethics committee of Wenzhou Medical University and its affiliated hospital.

### Sample collection and DNA extraction

Resections or biopsies of primary solid tumors and adjacent normal tissues located 2-cm away from the tumor tissue, were taken immediately after surgery, snap frozen in liquid nitrogen, and stored at − 80 °C. DNA was extracted from tissue using the QIAamp DNA mini kit (Qiagen, Hilden, Germany) according to the manufacturer’s instruction, and stored at − 20 °C for further use. The DNA concentration was quantified by NanoDrop One (Thermo Fisher Scientific, Foster City, CA, USA).

FFPE tumor samples were analyzed by hematoxylin-and-eosin (H&E) staining to determine tumor purity. For each FFPE tissue, 20 slices of 10-μm-thick sections were used for DNA extraction with the QIAamp DNA FFPE Tissue kit (Qiagen). Concentration of DNA was determined by a Qubit 3.0 Fluorometer (Thermo Fisher Scientific).

### CRC mutation panel design

The mutations were selected for either of the following three reasons: 1) present in at least two of the following three sources for CRC samples: TCGA (https://cancergenome.nih.gov/); International Cancer Genome Consortium (ICGC) database (https://icgc.org/); and publication by Muzny DM et al. [12]; 2) potential resistance to CRC targeted therapy based on My Cancer Genome (http://www.mycancergenome.org), or 3) reported in a commercial cancer panel [13].

The CRC mutation panel was designed with Assay Designer software (MassARRAY Typer, Version 4.0, Agena Biosciences, San Diego, CA, USA), with primer sequences not overlapping known single nucleotide polymorphisms whenever possible.

### CRC mutation detection and quantification by MALDI-TOF MS

CRC Mutation detection was optimized and performed by MassARRAY Analyzer 4 with CPM (Agena Biosciences). All primers were purchased from Integrated DNA Technologies (Integrated DNA Technologies, Coralville, IA, USA). All reagents were purchased from Agena Bioscience unless otherwise specified. Briefly, tissue genomic DNA was amplified by multiplex PCR. Shrimp alkaline phosphatase treatment was performed to inactivate surplus nucleotides. A primer extension reaction (iPLEX Pro) was performed with mass-modified terminator nucleotides, and the product was spotted on SpectroCHIP. Wild-type and mutant alleles were then discriminated by molecular weights determined by MassARRAY analyzer.

Allele calls were performed with MassARRAY Typer Analyzer software (Typer 4.0.26). Additionally, at least two investigators independently reviewed the mass spectra to further confirm the automated calls by the software. To estimate mutant allele frequency, the heights of raw spectral peaks corresponding to the wild-type and mutation allele were quantified. Mutation allele frequency was estimated by calculating mutant peak /(mutant peak + wild type peak).

### Targeted NGS and data analysis

Genomic DNA was fragmented to an average size of 200 to 500 bp using Bioruptor Pico sonication device (Diagenode, Denville, NJ, USA). Sequencing libraries were prepared using the KAPA LTP Library Preparation Kit for Illumina (Kapa Biosystems, Wilmington, MA, USA) according to manufacturer’s suggestions. Hybridization-based target enrichment was carried out with xGen Pan-Cancer Panel v2.4 (532 cancer-relevant genes), and xGen Lockdown Hybridization and Wash Reagents Kit (Integrated DNA Technologies). Captured libraries were amplified and purified using Agencourt AMPure XP Beads (Beckman Coulter, Atlanta, GA, USA). Concentrations and qualities of DNA libraries were analyzed by the Agilent High Sensitivity DNA Kit (Agilent Technologies, Santa Clara, CA, USA).

The libraries were paired-end sequenced on the Illumina HiSeq X platform (Illumina, San Diego, CA, USA) according to the manufacturer’s instructions. Sequencing adapters and low quality bases were trimmed from raw sequencing reads using Trim Galore (v0.4.1; https:// github.com/FelixKrueger/TrimGalore). The filtered reads were then mapped to the reference Human Genome (hg38) using BWA-MEM (v0.7.12; https://github.com/lh3/bwa/tree/master/bwakit) with the default settings. The GATK (v4.0.12.0; https://software.broadinstitute.org/gatk/) was used for single nucleotide variation (SNV) identifications. SNV calls with at least 2.5% variant allele frequency (VAF) were retained, followed by annotation using ANNOVAR [14].

### Mutation validation by capillary sequencing

A subset of samples were selected for Sanger sequencing to validate MS results. The capillary sequencing was performed with the BigDye Terminator Cycle Sequencing Kit and ABI 3730 Genetic Analyzer (Thermo Fisher Scientific).

### Statistical analysis

Statistical analyses were performed with IBM SPSS Statistics 20.0. The χ^2^ test or Fisher exact test were used to compare baseline categorical variables, and the Kruskal-Wallis test was used to analyze the association between mutation and tumor size. The Cox proportional-hazards model of multivariate analysis was performed to analyze covariables, such as age, gender, clinical stage, location, differentiation, metastasis, treatment after surgery, and tumor size.

## Results

### Patients information

A total of 300 patients diagnosed with colorectal cancer between July 2015 and June 2019 were recruited. Seventy one patients were excluded (see exclusion criteria in the methods section). Frozen tissues (paired tumor and adjacent normal tissues) from 136 patients, frozen tumor tissues from 47 patients, and formalin fixed paraffin embedded (FFPE) tissues (paired tumor and adjacent normal tissues) from 46 patients with CRC were analyzed. Table 1 summarizes the patients’ baseline characteristics, tumor stage, location, differentiation, size, follow-up treatment after surgery and tumor marker (CEA) level.

**Table 1:** Patient characteristics

Characteristic	All patients (***N*** = 229) (%)	Frozen tissue cohort (***N*** = 183) (%)	FFPE tissue cohort (***N*** = 46) (%)
**Age - no.**			
< 50	20 (8.7)	17 (9.3)	3 (6.5)
50–70	124 (54.1)	101 (55.2)	23 (50.0)
> 70	85 (37.2)	65 (35.3)	20 (43.5)
**Gender - no.**			
Male	145 (63.3)	113 (61.7)	32 (69.6)
Female	84 (36.7)	70 (38.3)	14 (30.4)
**Clinical stage - no.**			
I	53 (23.2)	34 (18.6)	19 (41.3)
II	69 (30.1)	59 (32.3)	10 (21.7)
III	82 (35.8)	71 (38.8)	11 (24.0)
IV	25 (10.9)	19 (10.4)	6 (13.0)
**Location sampled - no.**			
Rectum	177 (77.3)	140 (76.5)	37 (80.5)
Left Colon	19 (8.3)	17 (9.3)	2 (4.3)
Right Colon	21 (9.2)	16 (8.7)	5 (10.9)
Rectosigmoid Junction	12 (5.2)	10 (5.5)	2 (4.3)
**Differentiation - no.**			
Well	13 (5.7)	12 (6.6)	1 (2.2)
Moderate	180 (78.6)	145 (79.2)	35 (76.0)
Poor	28 (12.2)	19 (10.4)	9 (19.6)
Unknown^a^	8 (3.5)	7 (3.8)	1 (2.2)
**Metastatic sites - no.**			
Liver only	19 (8.3)	15 (8.2)	4 (8.7)
Lung only	2 (0.9)	1 (0.5)	1 (2.2)
Liver and Lung	1 (0.4)	1 (0.5)	0 (0.0)
Bladder	1 (0.4)	1 (0.5)	0 (0.0)
Brain	1 (0.4)	1 (0.5)	0 (0.0)
**Treatment after surgery - no.**			
With adjuvant therapy	129 (56.3)	109 (59.6)	20 (43.5)
Without adjuvant therapy	96 (42.0)	74 (40.4)	22 (47.8)
NA	4 (1.7)	0 (0.0)	4 (8.7)
**Tumor size - cm**			
Median (range)	4(1.5–13)	4 (1.5–13)	4(1.5–12)
**CEA - ng/mL**			
0.0–5.0	150 (65.5)	125 (68.3)	25 (54.3)
> 5.0	74 (32.3)	53 (29.0)	21 (45.6)
N/A	5 (2.2)	5 (2.7)	0 (0.0)

^a^Reported as mucinous adenocarcinomas

*N/A* not available

### Selection of somatic mutations and multiplex assay design

Somatic mutations for CRC were chosen based on their prevalence, and biological/clinical relevance. The final mutation panel consists of 299 mutations from 109 genes (Additional file 1: Supplementary Table 1).

**Fig. 1:**
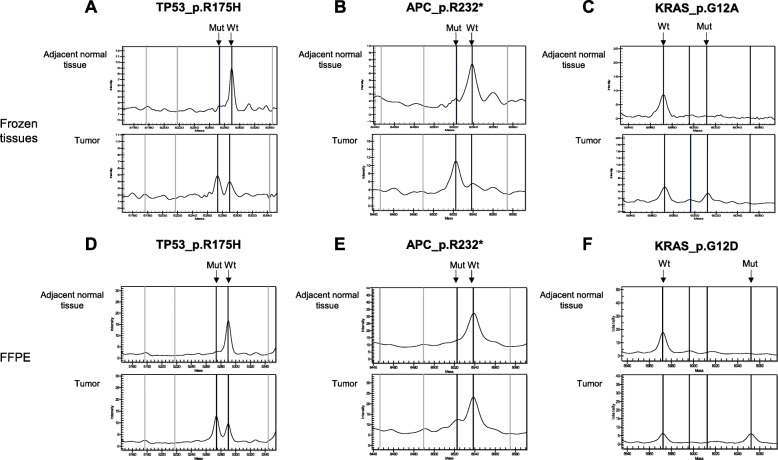
Representative MS results showing paired tumor and adjacent normal tissues from frozen and FFPE tissues. Shown in (**a**), (**b**), and (**c**) are three different mutations in the *TP53*, *APC* and *KRAS* genes from three frozen tissues, (**d**), (**e**) and (**f**) show the mass spectra for three FFPE tissues. Mutant allele frequency can be estimated by comparing the peak signals for the mutant and wild type alleles. Wt: wild type, Mut: mutation

We designed an 8-well multiple assay for a panel of 299 mutations (36-plex in well 1 and 2, 34-plex in well 3 and 5, 30-plex in well 4, 29-plex in well 6, 26-plex in well 7, and 12-plex in well 8) for MALDI-TOF MS analysis. The sequences of PCR primers and extension primers are provided in Additional file 1: Supplementary Table 2. Wild-type and mutant alleles were then discriminated by molecular weights of the extension products measured by MALDI-TOF MS (example results shown in Fig. 1).

**Fig. 2:**
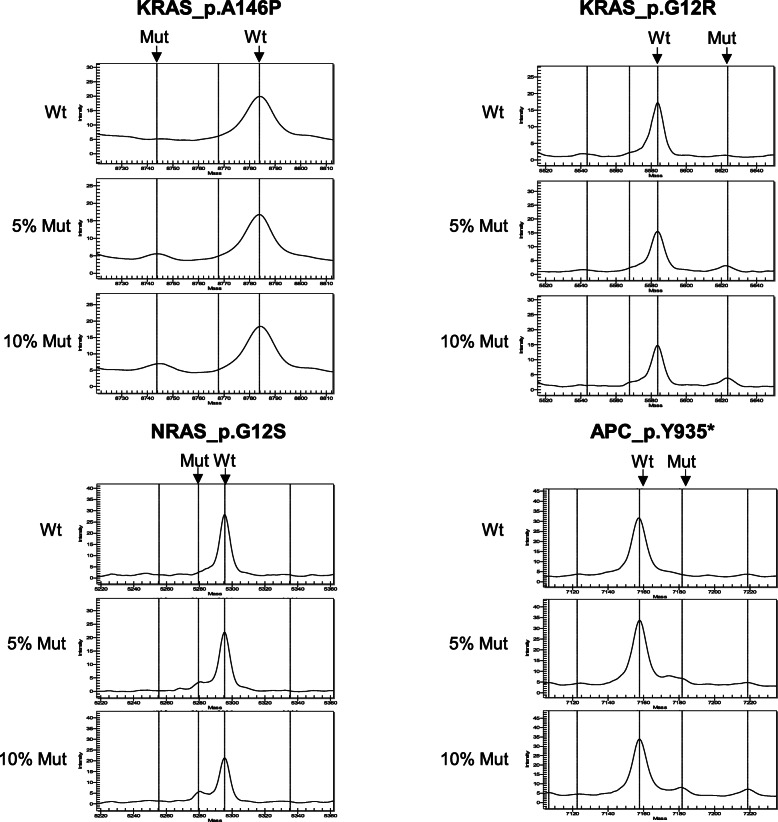
Evaluation the performance of the MS assay. The DNA mixture samples with 5 and 10% mutation were prepared to analyze the sensitivity and accuracy of the assay. Among them, 19 assays achieved a 5% sensitivity for mutations, and 16 assays achieved a 10% sensitivity. Here we show 4 assays that could achieve a 5% sensitivity

To determine the sensitivity of the assays, we constructed plasmids for 35 different mutations. Plasmid mutations were confirmed by Sanger sequencing. Mixtures of plasmids containing wild-type and mutant sequences to mimic samples with 5 and 10% mutation frequencies were prepared to analyze the sensitivity and accuracy of the assay. Among them, 19 assays achieved a 5% sensitivity for mutations, and 16 assays achieved a 10% sensitivity (Fig. 2 and Supplement figure 1).

### Mutation profiles of 229 patients from frozen and FFPE tissues

**Fig. 3:**
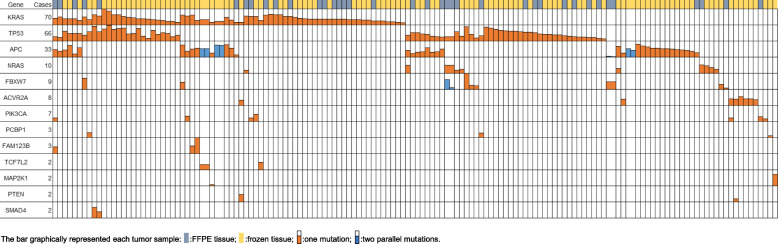
Mutation profiles quantified by the multiplex CRC panel. Each colored bar represents one mutation (orange) or two mutations in the same gene (blue). The heights of the colored bars represent mutant allele frequencies

Using the multiplex assay, we analyzed 411 samples from 229 patients (319 freshly frozen tissues from 183 patients and 92 FFPE samples from 46 patients) (Fig. 3). In the frozen tissue cohort, we identified and quantified 52 different somatic mutations in 107 patients. The most frequently mutated genes were *KRAS* (52 patients, 28.4%), *TP53* (48 patients, 26.2%) and *APC* (32 patients, 17.5%). We chose 20 mutations from 31 patients for Sanger sequencing validation. Sanger Sequencing showed complete concordance with MALDI-TOF MS (Additional file 2: Supplementary Figure 2).

In the cohort with 46 paired FFPE tissues (tumor and adjacent normal tissues), we first performed histopathological analysis with H&E staining (Supplement figure 3). With the multiplex MS assay, we detected a total of 39 mutations in 35 samples. Similarly, APC, TP53, and KRAS were most frequently mutated. We chose 15 different mutations (19 total mutations) from 13 patients for Sanger sequencing validation (Additional file 2: Supplementary Figure 4). Eighteen of the 19 mutations (95%) were validated successfully. Samples chosen for validation, as well as sequences of PCR primers and sequencing primers, are provided in Additional file 1: Supplementary Table 3.

Both frozen and FFPE tissues are commonly used for molecular pathology analysis. Tissue sampling differences may affect the somatic mutation frequencies in individual samples. For the three genes (*TP53*, *APC*, and *KRAS*) that are most commonly mutated in CRC, we found two genes (*TP53* and *APC*) showed higher mutation frequencies in the frozen tissues than the FFPE tissues. The average *TP53* mutation frequencies were 50.9 and 38.3% for the frozen tissues and FFPE tissues, respectively (*p* = 0.006, Mann Whitney U test). The average *APC* mutation frequencies were 47.5 and 26.8% for the frozen tissues and FFPE tissues, respectively (*p* = 0.005, Mann Whitney U test).

We next examined the association between the molecular alterations and patient characteristics (Table 2). Among the 142 patients with at least one somatic mutation identified, we found that the 63 patients without a *RAS* mutation exhibited different clinical stage distribution as compared with the 79 patients with *RAS* mutation (*p* = 0.019, χ^2^ test). *TP53* mutations were more frequently found in male patients by univariate analysis (*p* = 0.002, χ^2^ test).

**Table 2:** Patient characteristics in different molecular subgroups

	With any mutation	No mutation	***P*** value	With ***APC*** mutation	With other mutation but no ***APC*** mutation	***P*** value	With ***TP53*** mutation	With other mutation but no ***TP53*** mutation	***P*** value	With ***RAS*** mutation	With other mutation but no ***RAS*** mutation	***P*** value
**Patients - no.**	142	87		41	101		66	76		79	63	
**Age**												
Median (range) - yr	66 (40–90)	65 (31–87)		64 (42–90)	67 (40–82)		65 (44–82)	67 (40–90)		65 (42–88)	67 (40–90)	
Mean - yr	66.3	65.2		66.0	66.4		65	67.4		65.8	67.0	
< 50 - no.	11	9	0.508	5	6	0.845	5	6	0.185	5	6	0.517
50–70 - no.	77	47		21	56		40	37		46	31	
> 70 - no.	54	31		15	39		21	33		28	26	
**Gender - no.**			0.204			0.852			0.002			0.096
Male	85	60		24	61		49	36		43	42	
Female	57	27		17	40		17	40		36	21	
**Clinical stage - no.**			0.041			0.631			0.204			0.019
I	35	18		12	23		21	14		24	11	
II	37	32		12	25		18	19		13	24	
III	49	33		11	38		18	31		31	18	
IV	21	4		6	15		9	12		11	10	
**Location sampled - no.**			0.062			0.266			0.213			0.136
Rectum	117	60		35	82		57	60		70	47	
Left Colon	8	11		1	7		2	6		2	6	
Right Colon	9	12		1	8		2	7		3	6	
Rectosigmoid Junction	8	4		4	4		5	3		4	4	
**Differentiation - no.**			0.892			0.128			0.156			0.400
Well	8	5		0	8		5	3		6	2	
Moderate	113	67		36	77		50	63		60	53	
Poor	16	12		3	13		11	5		10	6	
Unknown^a^	5	3		2	3		0	5		3	2	
**Metastasis- no.**			0.017			1.000			0.815			0.814
With Metastasis	21	4		6	15		9	12		11	10	
Without Metastasis	121	83		35	86		57	64		68	53	
**Treatment after surgery - no.**			0.405			0.133			0.611			1.000
With adjuvant therapy	77	52		26	51		34	43		42	35	
Without adjuvant therapy	63	33		14	49		31	32		35	28	
N/A	2	2		1	1		1	1		2	0	
**Tumor size - cm**			0.332			0.066			0.598			0.479
Median (range)	4 (1.5–13)	4 (1.5–10)		4 (1.5–12)	4.3 (1.5–13)		4 (1.5–11)	4 (1.5–13)		4 (1.5–13)	4 (1.5–8.5)	
Mean	4.4	4.2		4.0	4.6		4.3	4.5		4.6	4.3	
**CEA - ng/mL**			0.057			0.703			0.491			1.000
0.0–5.0	87	63		26	61		39	48		49	38	
> 5.0	53	21		14	39		27	26		30	23	
N/A	2	3		1	1		0	2		0	2	

*P* values were calculated with the use of χ^2^ test or Fisher exact test for categorical variables and Kruskal-Wallis test for continuous variables

^a^ Reported as mucinous adenocarcinomas

*N/A* not available

### Comparison between targeted NGS and MS assays

We chose 13 patient samples and performed targeted NGS sequencing using xGen Pan-Cancer Panel V2.4. The NGS panel covers 532 genes. Deep sequencing was performed to have an average sequencing depth of 1400. Among these 13 patients, the MS method identified 11 somatic mutations. Targeted NGS detected 14 mutations, including the 11 mutations detected by MS. Three mutations were identified only by NGS. These three discordant mutations were further analyzed by DNA cloning and capillary sequencing. Two mutations (*KRAS*_c.G35A, *APC*_c.C2626T) were verified by cloning and sequencing. One mutation (*KRAS*_c.G34A) was present at 3% by targeted NGS. We sequenced 37 clones and did not observe any mutant.

## Discussion

We developed an 8-well, multiplexed assay based on automated MALDI-TOF mass spectrometry to detect 299 CRC related mutations in 229 southern China patients.

The selected 299 mutations cover the National Comprehensive Cancer Network (NCCN) guideline of colon cancer recommended gene list relevant to treatment and prognosis, such as *KRAS*, *NRAS*, *BRAF* V600E mutations [15–17]. The MALDI-TOF MS assay is sensitive and quantitative in analyzing somatic mutations, with extensive validation by capillary sequencing, as well as a head-to-head comparison with a targeted NGS panel covering 532 genes. We analyzed both frozen tissues and FFPE samples as both sample types are commonly used for molecular pathology analysis. In our cohorts, we found the frozen tissues may contain higher tumor content as evidenced by higher mutation frequencies for *TP53* and *APC*, which may be due to sampling differences.

NGS-based methods can detect known and unknown mutations while the MALDI-TOF MS assay is more suitable to detect pre-selected, functionally relevant known mutations. The cost of the multiplex MS assay (about USD 60/sample) is substantially lower than targeted NGS (about USD 150 ~ 200/sample). The MS approach is highly automated and suitable for high throughput analysis of larger sample size, making it suitable as a routine testing platform. The overall time needed for the MS assay is about 9 h, with hands-on time of about 60 min. The MS assay is also flexible in panel expansion to add more important mutations.

## Conclusions

We have developed and validated a highly multiplexed assay for the quantification of 299 somatic mutations in colorectal cancer tissues, offering a tool for studying the biological and clinical significance of somatic mutations with large numbers of cancer tissues. The multiplex assay may also be useful in clinical management of colorectal cancer patients.

## Supplementary information

**Additional file 1 Table S1**. Mutation list of CRC panel. **Table S2**. Primer sequences for mutation detection by CRC panel. **Table S3**. Primer sequences for mutation validation by Sanger sequencing. (XLS 145 kb)

**Additional file 2 Figure S1**. MS assays could detect 5 and 10% mutations mixed by wild-type and mutant plasmids. **Figure S2**. Genomic alterations of frozen tissues were detected by MALDI-TOF MS and confirmed by Sanger sequencing (A-F). **Figure S3**. Diagnosis and histopathological determination of Formalin Fixation and Paraffin Embedding tumor tissues (A-U) were conducted by analysis of H&E staining. **Figure S4**. Genomic alterations of FFTE tissues were detected by MALDI-TOF MS and confirmed by Sanger sequencing (A-F).

## Data Availability

The datasets used and analyzed during the current study are available from the corresponding author on reasonable request. All sequencing files are available from the NCBI BioProject database (https://www.ncbi.nlm.nih.gov/bioproject/PRJNA662695).

## Abbreviations

MALDI-TOF MS: Matrix-assisted laser desorption/ionization-time of flight mass spectrometry
PCR: Polymerase chain reaction
qPCR: Quantitative PCR
*KRAS*: KRAS proto-oncogene, GTPase
*TP53*: Tumor protein p53
*APC*: APC, WNT signaling pathway regulator
*EGFR*: Epidermal growth factor receptor
TKI: Tyrosine kinase inhibitor
*NRAS*: NRAS proto-oncogene, GTPase
NGS: Next-generation sequencing
TCGA: The Cancer Genome Atlas
CRC: Colorectal cancer
AJCC: American Joint Committee on Cancer
H&E: Hematoxylin-and-Eosin
ICGC: International Cancer Genome Consortium
SNV: Single Nucleotide Variation
SNPs: Single Nucleotide Polymorphisms
VAF: Variant Allele Frequency
CEA: Carcinoembryonic Antigen
*TCF7L2*: Transcription factor 7 like 2
PFS: Progress-free survival
NCCN: National Comprehensive Cancer Network
*BRAF*: B-Raf Proto-Oncogene, Serine/Threonine Kinase
