# Supplementary material for: Highly multiplexed quantifications of 299 somatic mutations in colorectal cancer patients by automated MALDI-TOF mass spectrometry
Source: BMC Med Genomics. 2020 Oct 2;13:143. doi: 10.1186/s12920-020-00804-y (PMC7532609; doi:10.1186/s12920-020-00804-y)
Supplement: Supplementary file 2 — Additional file 2 Figure S1. MS assays could detect 5 and 10% mutations mixed by wild-type and mutant plasmids. Figure S2. Genomic alterations of frozen tissues were detected by MALDI-TOF MS and confirmed by Sanger sequencing (A-F). Figure S3. Diagnosis and histopathological determination of Formalin Fixation and Paraffin Embedding tumor tissues (A-U) were conducted by analysis of H&E staining. Figure S4. Genomic alterations of FFTE tissues were detected by MALDI-TOF MS and confirmed by Sanger sequencing (A-F). [file 12920_2020_804_MOESM2_ESM.pdf]

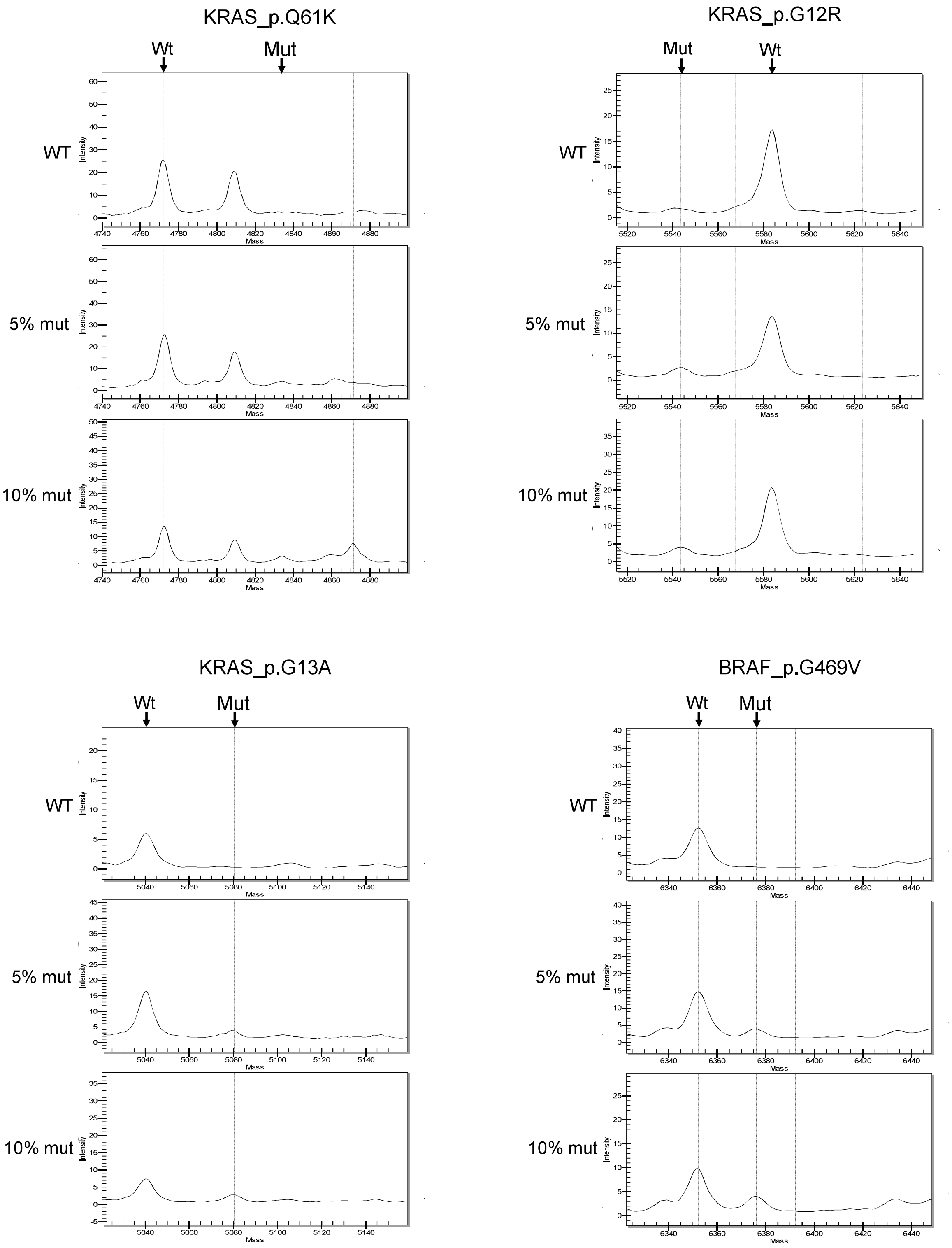

KRAS_p.Q61K
Wt
Mut
WT
5% mut
10% mut
Intensity
Mass
KRAS_p.G12R
Mut
Wt
WT
5% mut
10% mut
Intensity
Mass
KRAS_p.G13A
Wt
Mut
WT
5% mut
10% mut
Intensity
Mass
BRAF_p.G469V
Wt
Mut
WT
5% mut
10% mut
Intensity
Mass

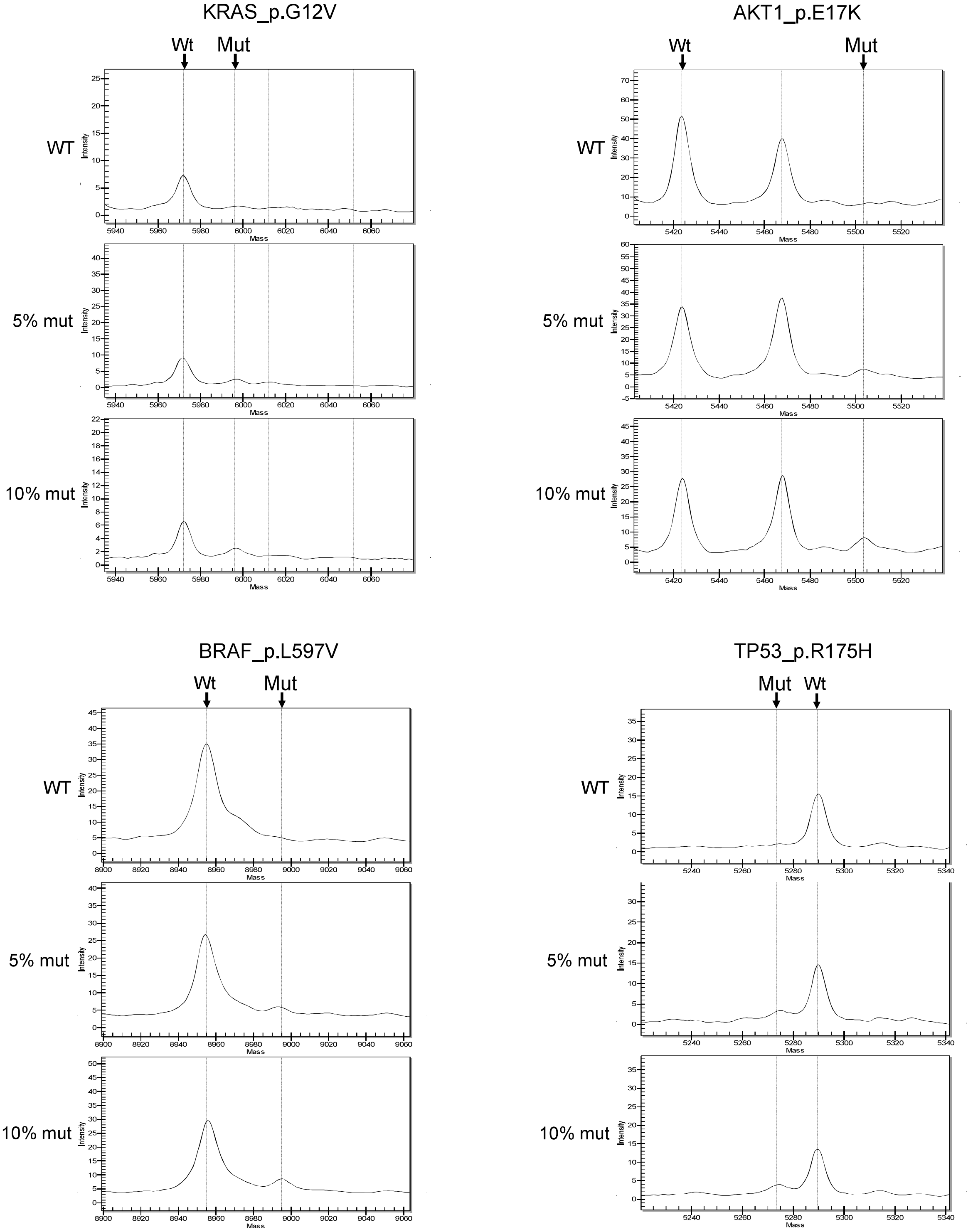

KRAS_p.G12V
Wt
Mut
WT
5% mut
10% mut
Intensity
Mass
AKT1_p.E17K
Wt
Mut
WT
5% mut
10% mut
Intensity
Mass
BRAF_p.L597V
Wt
Mut
WT
5% mut
10% mut
Intensity
Mass
TP53_p.R175H
Mut
Wt
WT
5% mut
10% mut
Intensity
Mass

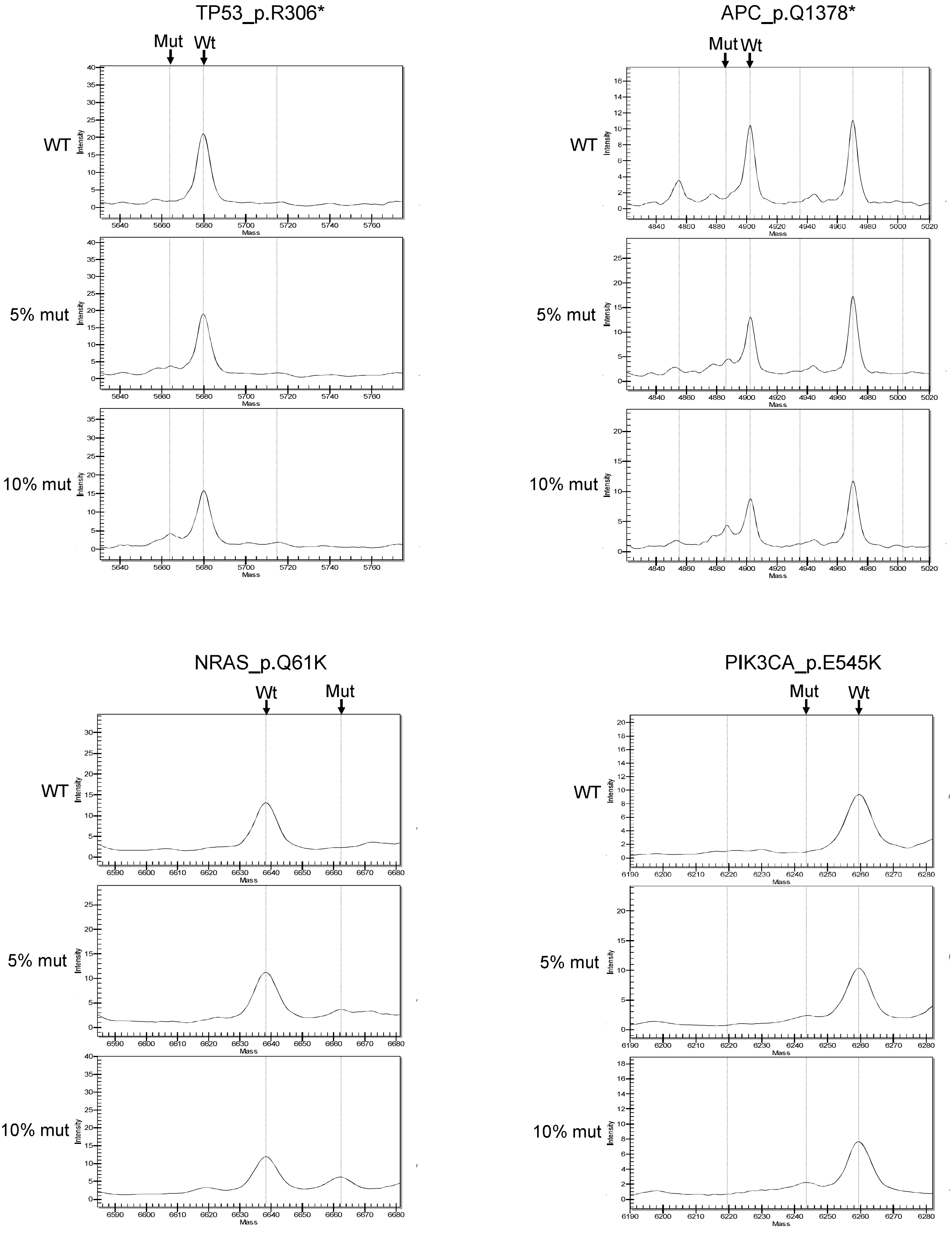
TP53_p.R306*
Mut
Wt
APC_p.Q1378*
Mut
Wt
NRAS_p.Q61K
Wt
Mut
PIK3CA_p.E545K
Mut
Wt
WT
5% mut
10% mut
Intensity
Mass

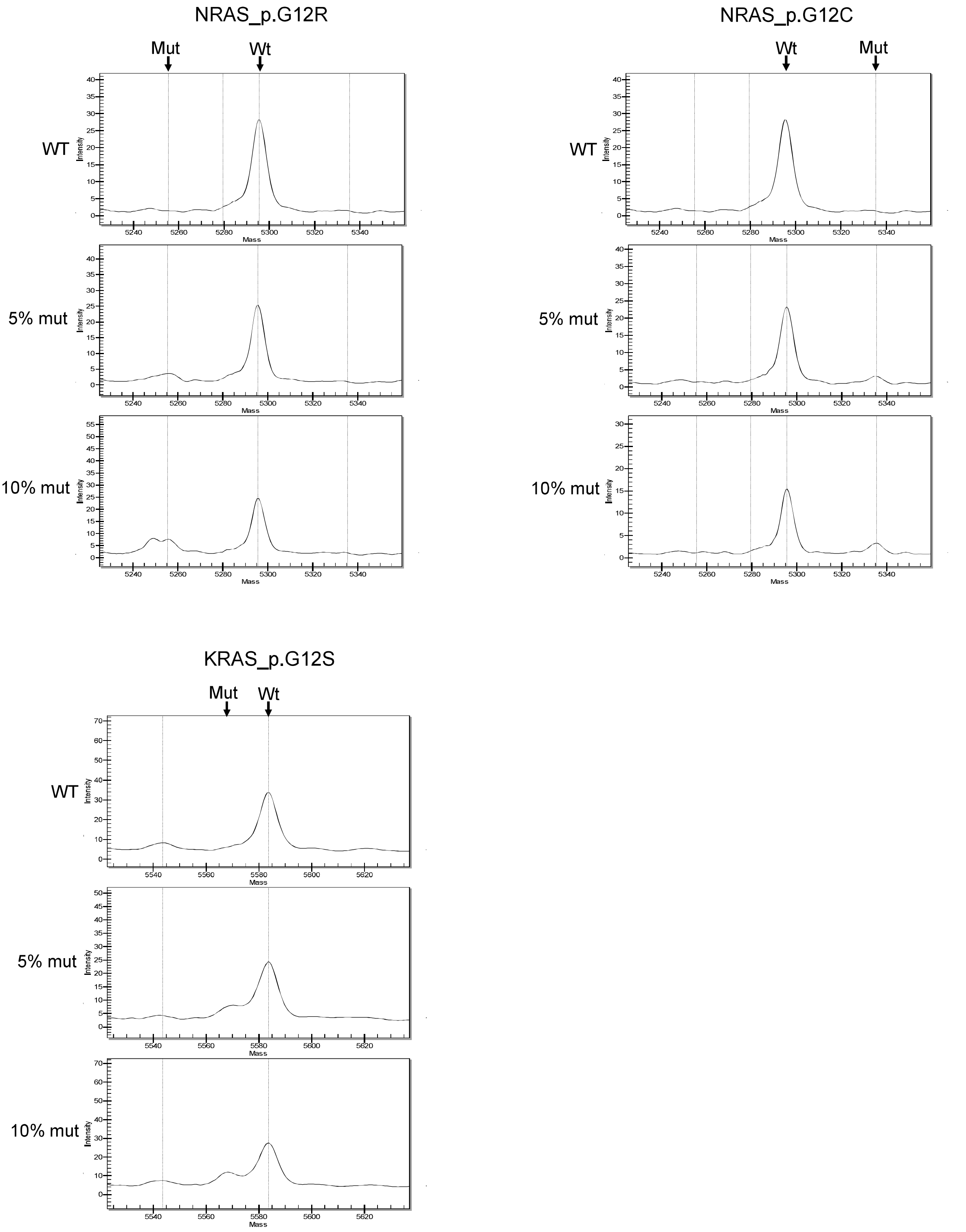

Supp Figure 1. MS assays could detect 5% and 10% mutations mixed by wild-type and mutant plasmids.

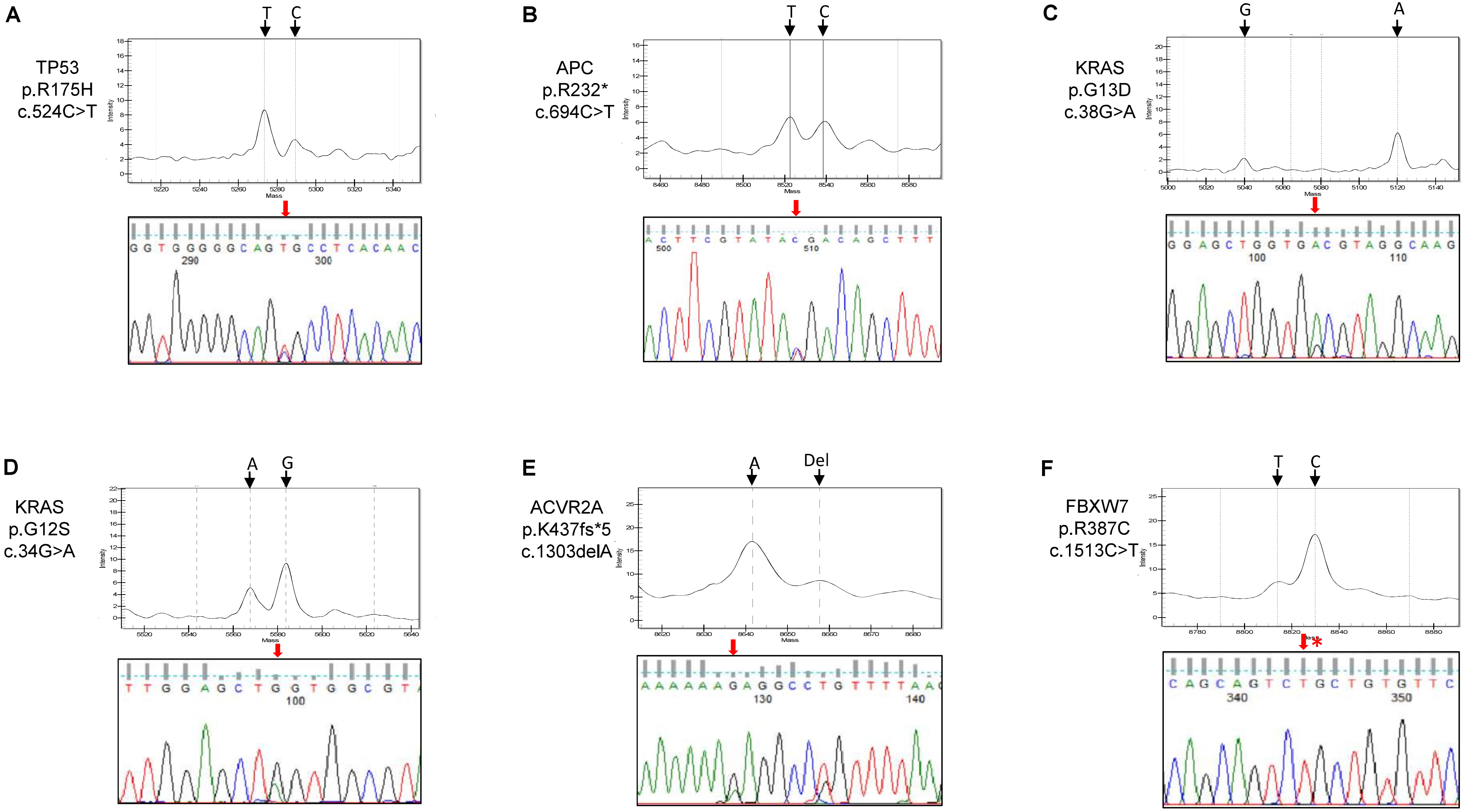

* one of eight clones (12.5%) was mutation T.

Supp Figure 2. Genomic alterations of frozen tissues were detected by MALDI-TOF MS and confirmed by Sanger sequencing (A-F).

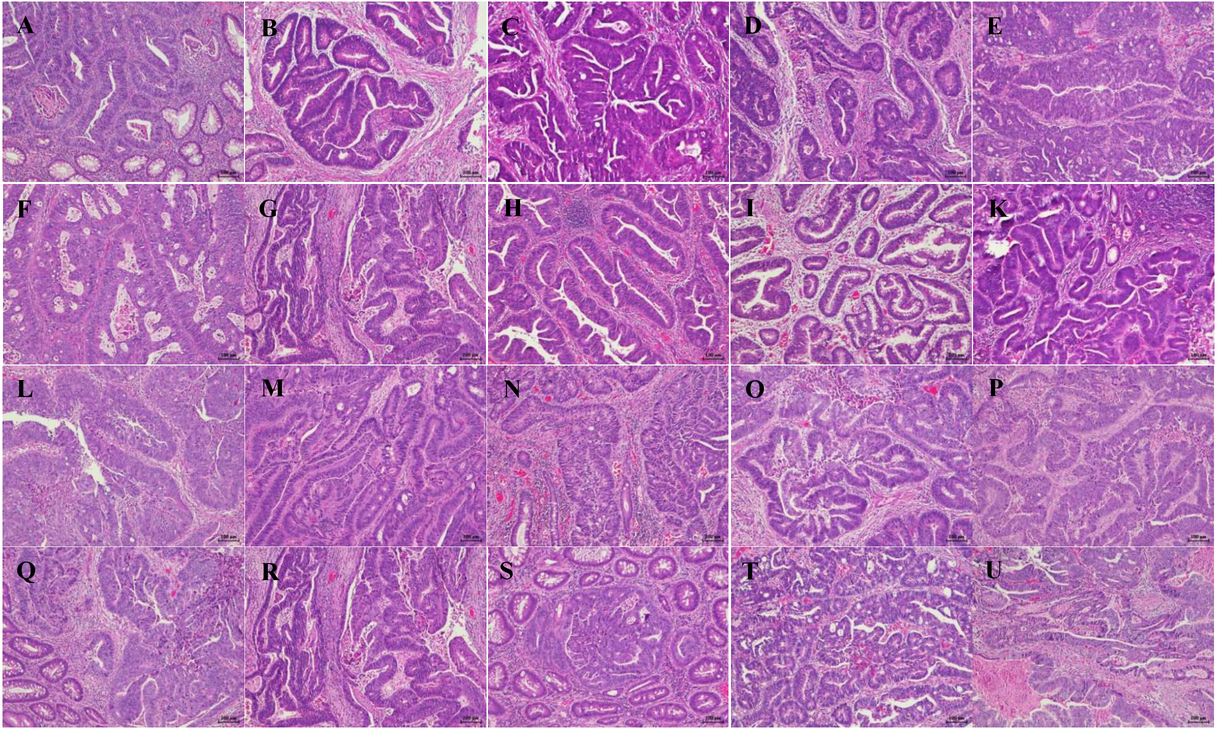

Supp Figure 3. Diagnosis and histopathological determination of Formalin Fixation and Paraffin Embedding tumor tissues (A-U) were conducted by analysis of H&E staining.

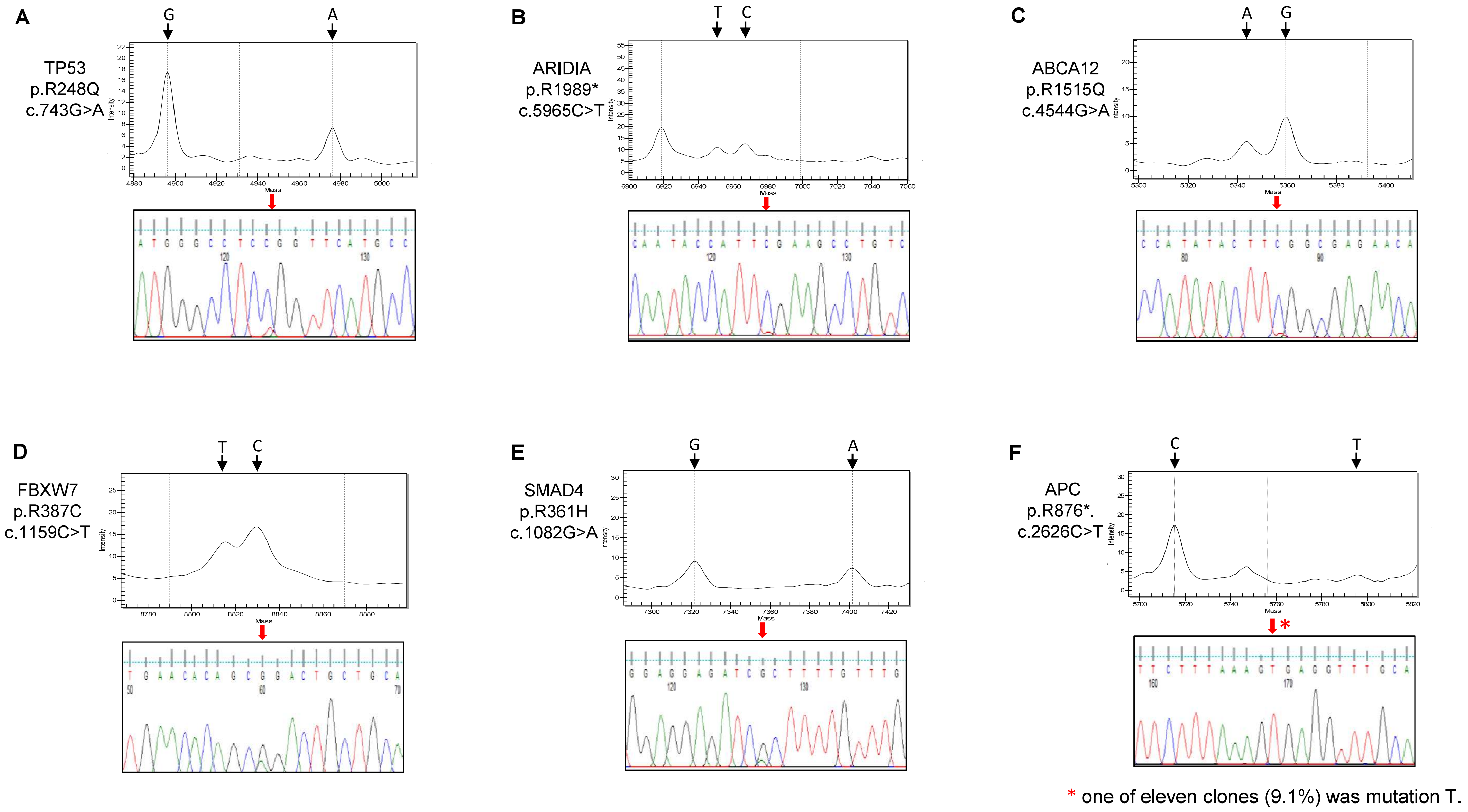

Supp Figure 4. Genomic alterations of FFPE tissues were detected by MALDI-TOF MS and confirmed by Sanger sequencing (A-F).
